# Platelet PD-L1 suppresses anti-cancer immune cell activity in PD-L1 negative tumors

**DOI:** 10.1038/s41598-020-76351-4

**Published:** 2020-11-09

**Authors:** Alexander B. Zaslavsky, M. P. Adams, X. Cao, T. Maj, J. E. Choi, J. Stangl-Kremser, S. Patel, A. Putelo, S. K. Lee, S. Nallandhighal, A. Kasputis, A. Alva, M. Lew, A. Qin, R. Mehra, T. M. Morgan, S. S. Salami, Z. Reichert, A. Udager, W. Zou, Ganesh S. Palapattu

**Affiliations:** 1 Department of Urology, University of Michigan Medical School, 3875 Taubman Center, 1500 E. Medical Center Dr., Ann Arbor, MI USA; 2 Rogel Cancer Center, University of Michigan, Ann Arbor, MI USA; 3 Department of Internal Medicine, Division of Hematology/Oncology, University of Michigan Medical School, Ann Arbor, MI USA; 4 Department of Surgery, University of Michigan Medical School, Ann Arbor, MI USA; 5 Michigan Center for Translational Pathology, University of Michigan Medical School, Ann Arbor, MI USA; 6 Department of Urology, Medical University of Vienna, Vienna, Austria; 7 Department of Pathology, University of Michigan Medical School, Ann Arbor, MI USA

**Keywords:** Cancer, Cancer microenvironment

## Abstract

Strategies that interfere with the binding of the receptor programmed cell death protein-1 (PD-1) to programmed death ligand-1 (PD-L1) have shown marked efficacy against many advanced cancers, including those that are negative for PD-L1. Precisely why patients with PD-L1 negative tumors respond to PD-1/PD-L1 checkpoint inhibition remains unclear. Here, we show that platelet-derived PD-L1 regulates the growth of PD-L1 negative tumors and that interference with platelet binding to PD-L1 negative cancer cells promotes T cell-induced cancer cytotoxicity. These results suggest that the successful outcomes of PD-L1 based therapies in patients with PD-L1 negative tumors may be explained, in part, by the presence of intra-tumoral platelets. Altogether, our findings demonstrate the impact of non-cancer/non-immune cell sources of PD-L1 in the tumor microenvironment in the promotion of cancer cell immune evasion. Our study also provides a compelling rationale for future testing of PD-L1 checkpoint inhibitor therapies in combination with antiplatelet agents, in patients with PD-L1 negative tumors.

## Introduction

Evasion of immune cell detection has been described as one of the hallmarks of cancer^1^. To survive and thrive in the tumor microenvironment, cancer cells have developed several mechanisms to subvert the host immune response^2^. One feature that has garnered recent attention is the ability of certain cancers to inactivate immune cells through checkpoint protein engagement^3, 4^. Programmed cell death protein 1 (PD-1) is a cell surface receptor present on T cells that once bound by its ligand, programmed death-ligand 1 (PD-L1), negatively regulates immune cell function^3,4^. In this way, cancer cells that acquire PD-L1 expression become resistant to cancer immune surveillance. Multiple studies have shown tumor tissue expression of PD-L1, and drugs that inhibit the interaction of this checkpoint pathway have demonstrated remarkable clinical efficacy in a variety of cancers (e.g., bladder, lung, kidney, and melanoma)^4,5^. Interestingly, however, the success of checkpoint inhibitor therapy that targets PD-L1 does not seem to be dependent on tumor PD-L1 expression^6–8^. To date, precisely how PD-L1 checkpoint pathway inhibitors exert antineoplastic effects in patients without tumor cell PD-L1 expression is not clear.

Cancer has been described as a wound that does not heal^9^. Although dysregulated, the same assortment of cells and molecular pathways triggered by the inflammatory response to injury are often found to be active within the tumor microenvironment^9,10^. Platelets are a major constituent of the body’s response to injury and have been shown to contribute to tumor growth and metastasis via the delivery of growth and angiogenic factors^10^. Platelets have also been shown to shield tumor cells from specific components of the immune response^11–15^. Notably, PD-L1 protein expression was recently reported in platelets of healthy individuals and patients with cancer^16^. The significance and role of platelet-derived PD-L1 on cancer associated immune cell activity, however, has not been described.

**Figure 1:**
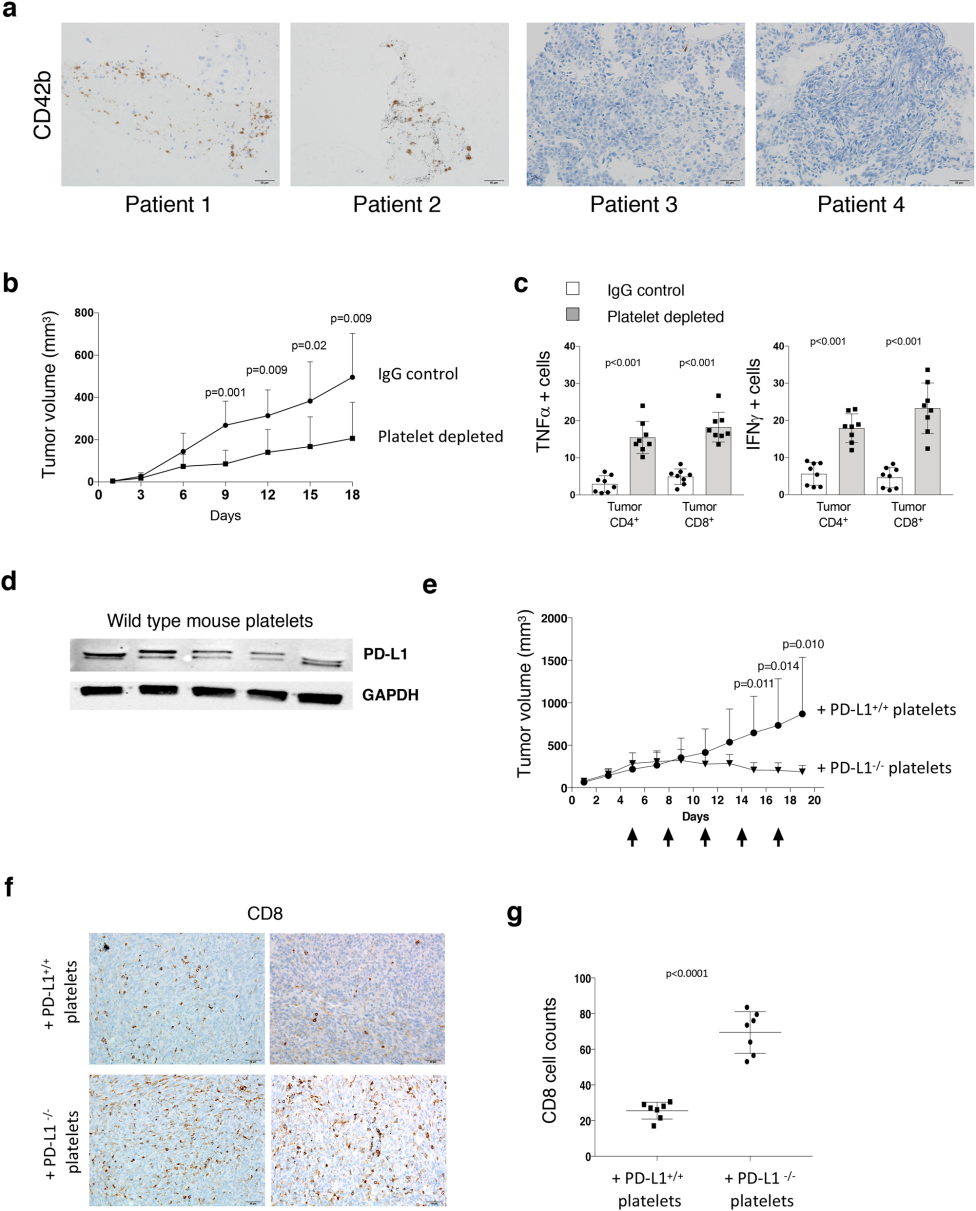
Platelet PD-L1 modulates PD-L1 negative tumor growth and T cell cytotoxicity. (**a**) Immunohistochemical analysis for platelets (CD42b) in human PD-L1 negative (metastatic lymph node) tumor tissues from two patients who did (#1 and #2) and (primary lung tissues) from patients who did not (#3 and #4) display a positive response to atezolizumab (anti-PD-L1 agent). Biopsy was taken before checkpoint inhibitor therapy was started. CD42b expression (brown). Scale Bar = 50 μm. (**b**) Wild type mice were inoculated with MC38 PD-L1 KO tumor cells and platelet depleted with antiplatelet GPIBα antibody or isotype control (IgG1). Tumor growth was measured by calipers at the indicated days. (**c**) Summary bar chart graphs show flow data from intracellular staining of MC38 PD-L1 KO tumor tissues. T cell (CD4^+^ and CD8^+^) effector cytokines (TNFα and IFNγ) were measured. Data are expressed as a percentage (n = 7 per group)**. **(**d**) Western blot of PD-L1 expression in wild type mouse platelets. Each lane in the blot represents PD-L1 protein expression in platelets from a single mouse. GAPDH is used as the loading control. **e.** PD-L1^−/−^ mice were inoculated with MC38 PD-L1 KO tumor cells. Mice were platelet depleted with antiplatelet GPIBα antibody before transfusion of platelets from wild type (PD-L1 positive) or PD-L1^−/−^ (PD-L1 negative) mice. Tumor growth was measured by calipers at the indicated days. Black arrows designate time points of platelet depletion and platelet transfusions. (**f**) Representative images of immunohistochemical staining of MC38 PD-L1 KO tumors from mice transfused with platelets from WT (PD-L1^+/+^) mice and PD-L1 KO (PD-L1^−/−^) mice with antibodies to CD8^+^ cell (brown) and Ki67 (brown) at 20X. Scale bar = 50 μm. (**g**) Summarizing bar graph showing the number of tumor infiltrated CD8^+^ cells per field for each MC38 PD-L1 KO tumor from mice transfused with platelets from either PD-L1^+/+^ (n = 8) or PD-L1^−/−^ mice (n = 8). Actual *p* values are shown unless non-significant (ns.). Students T-test (unpaired, two-tailed). Error bars represent the mean ± standard deviation.

To better understand the role of platelets in the response to checkpoint inhibitor therapy, we first assessed the presence of platelets in PD-L1 negative lung cancer tissues (lung and metastatic lymph node), obtained at biopsy pre-treatment, from patients who were treated with PD-L1 checkpoint blockade. We evaluated two patients who responded to anti-PD-L1 therapy and found an increased amount of intra-tumoral platelets as evidenced by prominent CD42b expression in comparison to PD-L1 therapy non-responders (Fig. 1a). These qualitative data suggested to us that platelet PD-L1 within the tumor microenvironment could be important in regulating an anti-tumor immune response and further motivated us to interrogate the influence of platelet PD-L1 on tumor-immune cell interaction and tumor growth in preclinical models.

Previous investigators have demonstrated that platelet depletion reduces tumor growth in mice^12,17–19^. Interestingly, recent studies have also demonstrated that co-culture of platelets with T cells results in decreased T cell production of IFNγ and TNFα in vitro^20^, and anti-PD-L1 therapy leads to increased numbers of activated T cells within tumors in animal models^21^. Here, to assess the effect of platelet PD-L1 on tumor growth, we depleted platelets in WT mice that had been previously subcutaneously inoculated with PD-L1 knockout (PD-L1 KO) murine colon adenocarcinoma MC38 cells. Tumor growth was monitored and platelet depletion was accomplished by serial (every 48 h) injection of either IgG (control) or a platelet-depleting antibody targeting platelet GPIBα. We found that tumor growth in platelet depleted mice was markedly diminished compared to controls (Fig. 1b and Sup. Fig. 1a). This finding was accompanied by significantly higher CD4^+^ and CD8^+^ cell production of IFNγ and TNFα in tumors from mice that were depleted of platelets compared to controls (Fig. 1c and Sup. Fig. 1b). These data suggest that tumor growth inhibition observed with platelet depletion is associated with increased intra-tumoral immune cell infiltration and activity.

To evaluate PD-L1 expression in mouse platelets, we isolated platelets from wild type (WT) mice. We observed that washed WT mouse platelets contain PD-L1 protein (Fig. 1d). To further interrogate the influence of platelet-derived PD-L1 on PD-L1 negative cancer cell growth, we inoculated PD-L1^−/−^ mice subcutaneously with PD-L1 knockout MC38 cells followed by platelet depletion via injection of a platelet-depleting antibody which targets mouse glycoprotein (GP) IBα (GPIBα) on platelets. Animals were then transfused (via tail vein) with washed platelets from either WT (PD-L1^+/+^) mice or PD-L1^−/−^ mice. Platelet depletions and platelet transfusions were repeated every 72 h as previously described^22^ until experiment termination. We observed that PD-L1^−/−^ mice transfused with platelets from PD-L1^+/+^ mice developed significantly larger tumors than PD-L1^−/−^ mice transfused with platelets from PD-L1^−/−^ mice (Fig. 1e and Sup. Fig. 1c). Next, to determine the effect of platelet PD-L1 on immune cell infiltration within tumors, we performed immunohistochemistry on excised MC38 PD-L1 KO tumors and observed a decreased number of tumor-infiltrating CD8^+^ cells in tumors from mice transfused with PD-L1^+/+^ mouse platelets (Fig. 1f,g). These data suggest that PD-L1 positive platelets promote PD-L1 negative tumor growth in PD-L1^−/−^ mice with reduced immune tumor cell infiltration.

**Figure 2:**
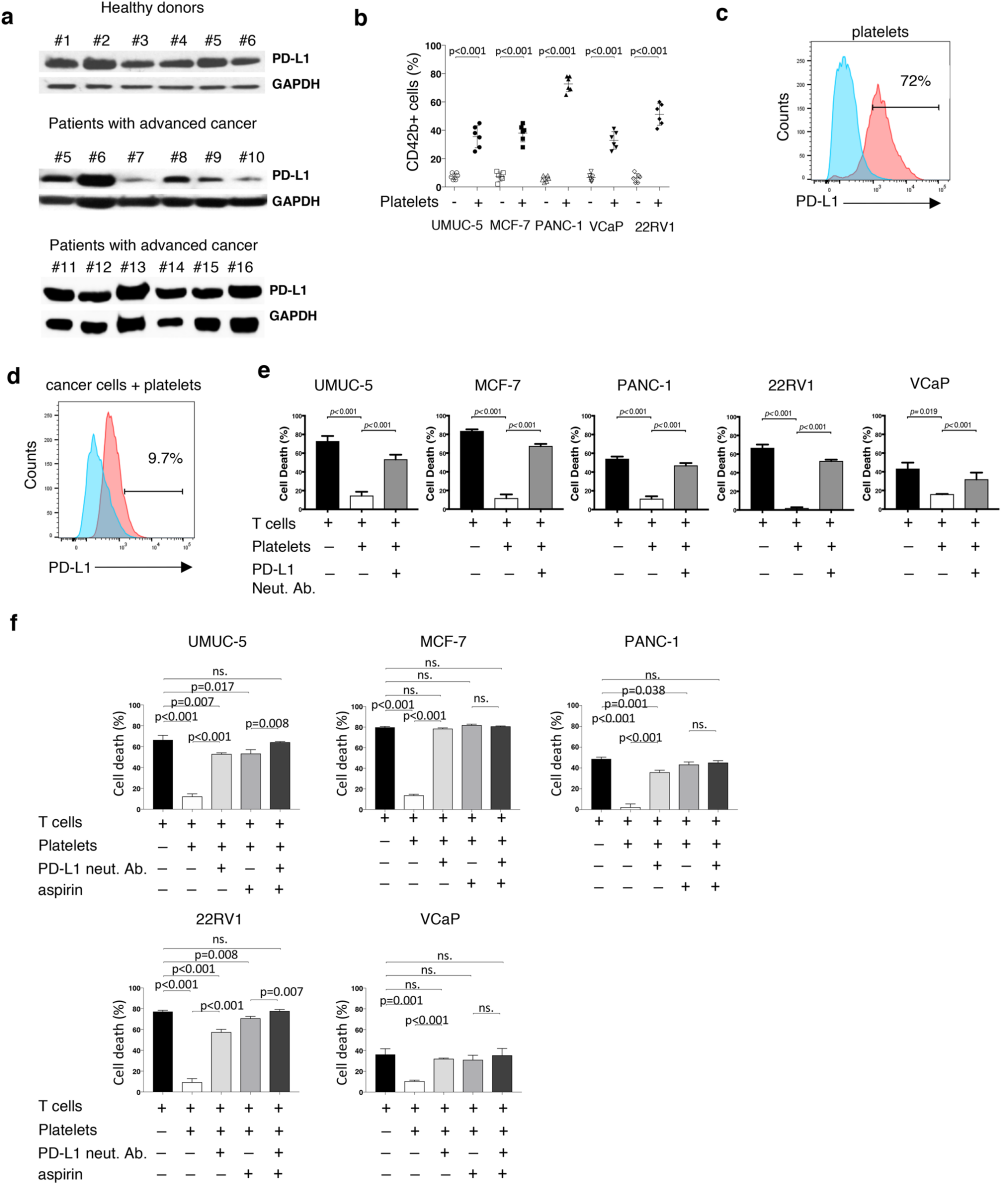
Blocking platelet adhesion to cancer cells increases T cell cancer cytotoxicity. (**a**) Western blot demonstrating PD-L1 expression in washed human platelets from healthy individuals (healthy donors) and patients with advanced cancer (Table 1). (**b**) Summarizing dot plot showing differential attachment of platelets (each dot indicates a single donor; n = 6) to tumor cells presented as percent tumor cells positive for platelet specific CD42b. (**c**) Representative flow cytometry histogram of a healthy donor (n = 5) platelet PD-L1 expression (blue) and following platelet activation with low dose thrombin (0.5 U/mL) (red). (**d**) Representative flow cytometry histogram of PD-L1 expression by PD-L1 negative UMUC-5 cancer cells before (blue peak) and after co-incubation with platelets from healthy donors (red peak) (n > 3). (**e**) T cell cytotoxicity and PD-L1 negative tumor cell lines. Tumor cells incubated with T cells only; Incubated with T cells in the presence of platelets; incubated with T cells in the presence of platelets and PD-L1 neutralizing antibody. The percent of dead cells is obtained from five independent sets of experiments using platelets from five different healthy donors. (**f**) T cell cytotoxicity and PD-L1 negative tumor cell lines. Tumor cells incubated with T cells only; Incubated with T cells in the presence of platelets; incubated with T cells in the presence of PD-L1 neutralizing antibody, and incubated in the presence of PD-L1 neutralizing antibody and aspirin. The percent of dead cells is obtained from five independent sets of experiments using platelets from five different healthy donors. Actual *p* values are shown unless non-significant (ns.). Students T-test (unpaired, two-tailed). Error bars represent the mean ± standard deviation.

To evaluate PD-L1 expression in human platelets, we isolated platelets from both healthy donor controls and patients with advanced cancer. We performed a Western blot and observed that washed platelets from all healthy controls and all patient samples contained PD-L1 although this was not quantified (Fig. 2a). We recognize that GAPDH protein may not be ideal loading control for platelets as its expression in platelets may be variable^23^. Next, to establish the expression levels of PD-L1 protein in cancer cells, we performed a Western blot using ten different human cancer cell lines including prostate, bladder, breast, and pancreatic cancer (Sup. Fig. 2). Previous reports have demonstrated the presence of platelet specific markers on the surface of human cancer cells following co-incubation, documenting platelet adhesion to cancer cells^15^. Hence, we performed flow cytometry analyses on five PD-L1 negative cell lines (UMUC-5, MCF-7, PANC-1, VCaP, 22RV1) following co-incubation with platelets from six healthy donors. These studies revealed the ability of platelets to bind to the surface of cancer cells (Fig. 2b).

**Table 1:** Basic characteristic features of studied patients.

Patient ID#	Gender	Age	Cancer type	Stage	Type of therapy
1	F	70	Lung adenocarcinoma	IV	Chemo., Atezolizumab
2	M	50	Lung squamous cell carcinoma	IIIA	Chemo., Atezolizumab
3	M	61	Lung adenocarcinoma	II	Chemo., Atezolizumab
4	F	52	Lung adenocarcinoma	II	Chemo., Atezolizumab
5	M	67	Urothelial carcinoma	IV	Chemo
6	M	64	Renal cell carcinoma	IV	Chemo
7	M	62	CRPC	IV	Chemo
8	M	68	CRPC	IV	Chemo
9	M	73	CRPC	IV	Androgen deprivation
10	M	69	CRPC	IV	Androgen deprivation
11	M	76	CRPC	IV	Chemo
12	M	79	CRPC	IV	Chemo
13	M	55	CRPC	IV	Chemo
14	F	57	Renal cell carcinoma	IV	Chemo
15	M	45	Renal cell carcinoma	IV	None
16	M	85	CRPC	IV	Androgen deprivation

Platelet activation has been previously demonstrated to be sufficient for the inactivation of T cells in vitro^20^. To ascertain if platelet activation is also associated with increased platelet PD-L1 expression we performed flow cytometry after activating platelets and found increased PD-L1 expression in platelets exposed to thrombin, an established platelet activator (Fig. 2c). Based on these findings, we hypothesized that platelet adhesion to PD-L1 negative cancer cells would result in the detection of PD-L1 protein on the cancer cell surface following platelet binding and activation. To test this, we co-incubated PD-L1 negative cancer cells with PD-L1 positive platelets from healthy donors and, after extensive washing to remove any unbound platelets, assessed tumor cell surface PD-L1 expression by flow cytometry. We observed that the addition of platelets to PD-L1 negative cancer cells resulted in the appearance of PD-L1 on the cancer cell surface (Fig. 2d and Sup. Fig. 3a,b). To explore this further, we performed Western blot, after extensively washing the cells to remove non-adherent platelets. We found that following platelet co-incubation, all PD-L1 negative cancer cells were positive for PD-L1 protein expression (Sup. Fig. 3c).

To establish whether platelet-derived PD-L1 protein is sufficient to inactivate immune cell function, we adopted a previously described method for PD-1/PD-L1 pathway interaction studies^24^ and performed cytotoxicity assays utilizing Jurkat T cells in the presence or absence of PD-L1 neutralizing antibody. We found that platelets protected against T cell induced cancer cell cytotoxicity and that this effect could be partially rescued with the addition of PD-L1 neutralizing antibody (Fig. 2e).

Next, we sought to study platelet binding to cancer cells and performed flow cytometry analyses on PD-L1 negative cancer cells following co-incubation with the anti-platelet agent aspirin. We observed significantly reduced, but not eliminated, platelet-cancer cell attachment with aspirin exposure (Sup. Fig. 4a,b). To investigate if T cell induced cytotoxicity could be enhanced in the presence of anti-platelet agents, we performed cytotoxicity assays in the presence of platelets, aspirin, and PD-L1 neutralizing antibody. We observed that combinatorial treatment, comprised of aspirin and PD-L1 neutralizing antibody, significantly improved T cell mediated cytotoxicity in all human cancer cell lines evaluated (Fig. 2f). Importantly, we found that in two PD-L1 negative cancer cell lines (UMUC-5 and 22RV1) the addition of aspirin significantly improved PD-L1 neutralizing antibody efficacy.

In the present study, we investigated the role of platelet PD-L1 as a potential modulator of tumor growth and T cell associated cancer cell cytotoxicity. Principally, we found that platelets expressing PD-L1 can attach to tumor cells and in this way modify the cancer cell associated immune response. Specifically, we found that platelet depletion in wild type animals resulted in significant inhibition of PD-L1 negative tumor growth and that this was associated with an increase in activated CD4^+^ and CD8^+^ cells within the tumor. Furthermore, we found that the growth of PD-L1 negative tumors in PD-L1^−/−^ mice was dramatically augmented following transfusion with PD-L1 positive platelets. We also observed, in vitro, that the inhibitory effect of platelets on T cell mediated cytotoxicity could be mitigated by the addition of the anti-platelet agent aspirin. Lastly, we observed a preponderance of platelets within PD-L1 negative tumors from patients that responded to PD-L1 checkpoint targeted therapy, suggesting an important role for platelet PD-L1, and possibly other non-immune cell sources of PD-L1, in immuno-oncology. Taken together, our findings provide insight into the successful outcomes of PD-L1 based therapies in patients with PD-L1 negative tumors and present the basis for the testing of antiplatelet agents in combination with checkpoint inhibitors, particularly in patients with PD-L1 negative tumors (Sup. Fig. 5).

Over the past several years, platelets have gained attention as important players in cancer growth and metastasis. Platelet-cancer cell binding leads to platelet activation and the selective release of platelet stored factors^14^. Exposure of cancer cells to platelet content has been linked to changes in tumor cell gene expression and changes within the tumor microenvironment^25,26^. Intriguingly, there has been recent interest in investigating the role of platelets as a component of the immune system^27,28^. Earlier work by Placke et al.^15^ demonstrated that platelet-cancer cell binding results in the transfer of MHC1 protein complex from platelets to cancer cells sufficient to inhibit NK cell activation. This important study demonstrated how cancer cells may utilize platelets in the tumor microenvironment to evade immune cell detection. Additionally, a more recent work utilizing an animal model found that platelet-derived TGFβ released upon platelet binding to cancer cells is capable of inactivating T cells^13^. Our experiments validate and expand on previous observations that platelets modulate the immune system to support cancer growth and implicate platelet PD-L1 as operational in this process. We did not investigate the interaction of intratumoral platelets and PD-1 expression. Further studies are warranted to determine the influence of platelets in this scenario. Future work is also needed to fully understand the role of non-cancer cell derived PD-L1 in creating and sustaining the immune suppressive tumor microenvironment and to uncover novel therapeutic targets that have the potential to improve the efficacy of immunotherapeutic strategies.

## Methods

### Human platelet isolation

Written informed consent in accordance with the Declaration of Helsinki was received from all participants before inclusion in the study. All patient samples were obtained under an institutional review board-approved protocol at the University of Michigan following informed consent. Healthy donor controls were healthy men and women, (25–70 years old), without known cancer and with no history of taking anti-platelet or anticoagulant medications. Whole blood was collected using 0.109 M sodium citrate as an anticoagulant. Separation of platelets from whole blood was achieved by centrifugation, as previously described^29^.

### In vivo mouse models

Eight-week-old wild type mice were obtained from Jackson Laboratory. PD-L1^−/−^ mice were obtained as previously described^21^. All mice were maintained under pathogen-free conditions. For MC38 tumor models, 1 × 10^6^ tumor cells were subcutaneously injected between the mouse shoulder blades. Wild type mice were platelet depleted by intravenous administration of 4 μg/g anti-mouse GPIb (R300, Emfret analytics, Germany) on day 3 after cancer cell inoculation and then every 72 h for the duration of the experiment. IgG_2a_ isotype Ab (MedImmune) was given intravenously at a dose of 100 μg per mouse to the control group. PD-L1^−/−^ mice were platelet depleted and transfused with 4 × 10^8^ WT or PD-L1^−/−^ mouse platelets as previously described^22^ once tumors reached 300 mm^3^ Tumor diameters were measured using calipers every other day. Tumor volumes were calculated using formula (W^2^ × L) × 5/9. All animal experiments were approved by the Animal Welfare Committee of the University of Michigan and were performed in accordance with federal and local guidelines and regulations.

### Flow cytometry analysis

Single-cell suspensions were prepared from fresh mouse tumor tissues by slicing tissues with a scalpel into small pieces, minced with a 23 Ga needle in cold DMEM medium supplemented with 10% FBS, followed by passing cells through a 100-μm filter. Tumor tissue cells were then centrifuged and resuspended in FACS buffer (D-PBS supplemented 1% BSA) before being stained with specific antibodies against mouse CD45 (30-F11), CD90 (53-2.1), CD4 (RM4-5), CD8 (53-6.7). T cell cytokine expression was determined by intracellular staining, using antibodies against mouse IFNγ (XMG1.2), and TNFα (MP6-XT22). All flow samples were analyzed on LSR II (BD), and data were analyzed with DIVA software (BD Biosciences) or FlowJo (version 10) flow cytometry software (FlowJo LLC, Ashland, OR).

To analyze platelet adhesion to cancer cells Washed platelets were adjusted to a concentration of 3 × 10^8^ platelets/ml in Tyrode’s Buffer (134 mM NaCl, 12 mM NaHCO_3_, 2.9 mM KCl, 0.34 mM Na_2_HPO_4_, 1 mM MgCl_2_, 10 mM HEPES, pH 7.4.). Platelets were then incubated with 3 × 10^5^ cancer cells for 20 min at 37 °C in FACS Buffer (D-PBS (Gibco; Life Technologies), supplemented with 1% heat-inactivated FBS). Cancer cells were washed to remove non-attached platelets twice with D-PBS; resuspended in 100 μl FACS Buffer and stained with 5 μl of CD42b antibody (APC Mouse Anti-Human CD42b, (BD Pharmingen, Cat. No. 551061) or with 5 μl of human PD-L1 antibody (BD Pharmingen, Cat. No. 557924) for 20 min at room temperature in the dark. Cells were washed once in FACS Buffer and then cell pellets were resuspended in 300 ml FACS Buffer before being transferred to flow tubes for flow cytometry analysis using BIORad ZE5 Cell Analyzer. Data were analyzed using FlowJo (version 10) flow cytometry software (FlowJo LLC, Ashland, OR).

### Cell culture

Mouse colon cancer cell line MC38 PD-L1 knockout cell line was a kind gift from Dr. W. Zou’s lab. Human cancer cell lines PANC-1 (pancreatic cancer) and prostate cancer cell lines 22RV1, DU145, VCaP, and PC-3 were obtained from the American Type Culture Collection (Manassas, VA). Jurkat (human T cells) were obtained from the American Type Culture Collection (Manassas, VA). Luciferase positive human bladder cancer cell lines UMUC-5 and UMUC-6 were a kind gift of Dr. M. Day (University of Michigan). Human breast cancer cell lines MCF-7 and MDA-MB231 cell lysates were obtained from the laboratory of D. Hayes (University of Michigan). The human lung cancer cell lysate H358 was provided by the lab of S. Tomlins (University of Michigan). 22RV1 and PC-3 cells were maintained in RPMI 1640 medium (Gibco; Life Technologies) supplemented with 10% heat-inactivated FBS, 2 mM l-glutamine, 100 U/ml penicillin G sodium, and 100 μg/ml streptomycin sulfate. MC38 PD-L1 KO, DU145, VCaP, PANC-1, MCF-7, UMUC-5 and UMUC6 cells were maintained in DMEM (1×) + GlutaMAX-1, 4.5 g/l D-Glucose, 110 mg/l Sodium Pyruvate (Gibco; Life Technologies) supplemented with 10% heat-inactivated FBS, 100 U/ml of penicillin–streptomycin (Thermo Fisher Scientific, Cat. No. 15070063). Jurkat cells were maintained in RPMI 1640 medium (Gibco; Life Technologies) supplemented with 10% heat-inactivated FBS. All cells were authenticated by the University of Michigan DNA Sequencing Core using short tandem repeat DNA fingerprinting. All cell lines were regularly examined for mycoplasma contamination.

### Generation of luciferase positive cells

Luciferase positive cells were generated by retroviral infection of the recombinant retrovirus vector obtained from the University of Michigan Vector Core (Lenti-GF1-CMV-VSVG). Luciferase cell expression was confirmed 72 h post infection using the IVIS200 system (Invitrogen).

### Bioluminescent cellular cytotoxicity with jurkat

Platelets were harvested from whole blood as described above and incubated either with PD-L1 neutralizing antibody (BPS Bioscience, Cat. No. 71213) at 20 μg/ml for 10 min at room temperature or with acetylsalicyclic acid (i.e. aspirin) at 18 μg/ml (Sigma, Cat. No. A5376) in Tyrode’s Buffer for 20 min at 37 °C. Cancer cells were incubated at 5000 cells per 100 μl of 2% RPMI with platelets at 3 × 10^8^ platelets/ml and seeded in 96 well plates (Falcon, Cat. No. 353296). Designated cancer cells were then co-incubated with Jurkat at 1:2 (target:effector) ratios based on our initial cytotoxicity assay results (Supplemental Fig. S1) for 6 h in the cell culture incubator. Before the next step, the 96 well plates were centrifuged for 3 min at 1200 RPM before the supernatant was removed by plate inversion over the laboratory sink. The remaining attached cells were then treated with 20 μl of 1 × Passive Cell Lysis Buffer (Promega), followed by additional 5 min incubation at room temperature. Luminescence was measured within 10 min using infinite M1000Pro (Application: Tecan i-control, 1.10.4.0) after the addition of 100 μl of One-Glo EX Luciferase Assay Substrate in One-Glo EX Luciferase Assay Buffer (Promega, Cat. No.E8130).

### Inhibition of platelet binding with aspirin and immunoblotting

Cancer cells were detached using 0.25% Trypsin-EDTA (1×) (Gibco; Life Technologies, Cat. No. 25200-056). Cancer cells were then incubated with and without platelets, and platelets pre-incubated with aspirin for 20 min at room temperature at 18 μg/ml. Cancer cells and platelets were co-incubated for 20 min in a cell culture incubator at 37 °C. followed by centrifugation for 3 min at 1000 RPM to pellet only tumor cells with attached platelets in 15 mL Falcon tubes (Fisher Scientific). The supernatant was removed by vacuum and cells were washed 2× in Tyrode’s Buffer with additional centrifugation after each wash for 3 min at 1000 RPM. The supernatant was removed after each wash. Cell pellets composed of cancer cells with attached platelets were then lysed in RIPA buffer. Protein concentration was measured against albumin standards (Thermo Scientific) in 1 × Bio-Rad Protein Assay Dye Reagent (Cat. No. 5000006). Protein samples were then mixed 1:1 with Laemmli Sample Buffer (Bio-Rad, Cat. No. 161-0737) and 2-Mercaptoethanol (Sigma, Cat. No. M3148). 125 μg of each protein sample was loaded into the wells of 12% gradient Bio-Rad Mini-Protean TGX Gel (Cat. No. 456-1043). Samples were run in running buffer, 1× Tris/Glycine/SDS buffer (Bio-Rad, Cat. No. 161-0732) at 110 V for 70 min before being transferred onto nitrocellulose membranes using transfer buffer, 1× NuPage (Novex; Life Technologies, Cat. No. NP0006-1) and 20% methanol, at 110 V for 70 min. Membranes were incubated with anti-PD-L1 antibody (Cell Signaling Technologies, Cat. No. 13684), or anti-GAPDH antibody (Millipore Sigma, Cat. No. 3005273) followed by secondary antibodies.

### Immunohistochemistry

Staining for mouse CD8 (CD8a D4W2z, Cell Signal. #98941) was performed on paraffin embedded tumor sections. T cells were counted manually at 20 high-power fields under a light microscope (Olympus). Immunostaining on human paraffin embedded cancer tissues was performed with the rabbit CD42b antibody (EPR6995, Abcam). Appropriate negative (Bone marrow) and positive controls (spleen) were stained in parallel with each set of slides studied.

### Human tumor tissues

We have an IRB-approved study to retrospectively study patients (clinical characteristics and associated tumor tissue) with advanced non-small cell lung cancer (NSCLC) treated with an anti-PD-1/PD-L1 agent at the University of Michigan (protocol #HUM00161860). For our analyses, archival tissue at the time of diagnosis was used in patients with stage IV non-small cell lung cancer were used. All were treated with atezolizumab (anti-PD-L1 agent) as part of the standard of care. Pretreatment biopsy was taken from either primary lung tissue or metastatic lymph node.

### Statistical analysis

GraphPad Prism software (GraphPad Software, Inc.) (v.7) was used for all statistical analyses. Pairwise significance was calculated with an unpaired, two-tailed *t* test. Actual *p* values are shown, unless *p* showed no significant difference, marked as non-significant (ns.).

## Supplementary information

Supplementary Legends.

Supplementary Figures.
