# Supplementary material for: Platelet PD-L1 suppresses anti-cancer immune cell activity in PD-L1 negative tumors
Source: Sci Rep. 2020 Nov 9;10:19296. doi: 10.1038/s41598-020-76351-4 (PMC7652857; doi:10.1038/s41598-020-76351-4)
Supplement: Supplementary file 1 — Supplementary Legends. [file 41598_2020_76351_MOESM1_ESM.docx]

**Supplemental Figures**

**Supplemental Figure 1. of MC38 PD-L1^-/-^ tumors. Histologic tumor images and flow cytometry data of intracellular staining.**  **a.** Tumor images from platelet depleted wild-type mice. **b.** Representative original flow cytometry data of intracellular staining of MC38 PD-L1^-/-^ tumor tissues. T cell (CD4^+^ and CD8^+^) effector cytokines (TNFα and IFNγ) were measured. Data are expressed as a percentage (n = 7 per group). **c**. Tumor images from platelet depleted PD-L1^-/-^ mice that were transfused with platelets from wild type (PD-L1 pos.) (n=8) or PD-L1^-/-^ (PD-L1 neg.) mice (n=8). **d.** Gating strategy for cytokine expression in T cells. The cells were initially gated based on size and granularity. The events with high forward scatter width versus forward scatter area were rejected as doublets. CD90 was used as a T cell marker, and gated further based on CD4 and CD8 expression. Each of these populations was further analyzed for IFNγ and TNFα expression as shown in the final step.

**Supplemental Figure 2. PD-L1 protein expression by cancer cells.** Western blot showing PD-L1 expression in various human cancer cell lines.

**Supplemental Figure 3. Platelet binding to cancer cells. a.** Representative flow cytometry histograms of cell surface PD-L1 expression of PD-L1 negative cells after incubation with platelets. The experiment was performed three times for each cell line using platelets from different healthy donors. **b.** Gating strategy to assess platelet-derived PD-L1 on PD-L1 negative cells after co-incubation with platelets. Cancer cells were initially gated based on size and granularity. The events with high forward scatter width versus forward scatter area were rejected as doublets. The singles were then evaluated for the PD-L1 expression as shown in the final step. **c.** Western blot of PD-L1 in cancer cells alone and in cancer cells after co-incubation with platelets.

**Supplemental Figure 4. Effect of aspirin on platelet binding to cancer cells. a.** Flow cytometry analyses depicting cancer cells positive for the presence of platelet marker (CD42b) on the cancer cell surface following platelet-cancer cell incubation with aspirin (upper 3 panels) or in its absence (lower 3 panels). **b.** Summarizing dot plot showing the effect of aspirin on platelet (n=3 platelets from different healthy controls) attachment to cancer cells following co-incubation. Data presented as fold change in the percent of cells stained positive for platelet marker in the presence of aspirin.

**Supplemental Figure 5.** **Proposed model for the impact of PD-L1 positive platelets on PD-L1 negative tumor growth.** **a.** Activated T cells may be capable of controlling PD-L1 negative tumor growth in the absence of the tumor cell bound platelets. **b.** Binding of PD-L1 positive platelets to some PD-L1 tumor cells may support tumor growth by interfering with intratumoral T cell activation. **c.** PD-L1 inhibitor suppresses PD-L1 negative tumor cell growth by interfering with platelet ability to inactivate intratumoral T cells.
