# Supplementary material for: Platelet PD-L1 suppresses anti-cancer immune cell activity in PD-L1 negative tumors
Source: Sci Rep. 2020 Nov 9;10:19296. doi: 10.1038/s41598-020-76351-4 (PMC7652857; doi:10.1038/s41598-020-76351-4)

Platelet PD-L1 suppresses anti-cancer immune cell activity in PD-L1 negative tumors

Zaslavsky A.[1,2*], Adams M.P.[1,2], Cao X.[1,2], Maj T[3]., Choi JE[2],[4], Judith Stangl-Kremser[1,6], Patel S.[1,2], Putelo A.[1,2], Lee S.[1,2], Nallandhighal S.[1,2], Kasputis A.[1,2], Alva A.[2,3], Lew M[7]., Qin A.[3], Mehra R.[7], Morgan T.M.[1,2], Salami S.[1,2], Reichert Z.[2,3], Udager A.[7], Zou W.[4], Palapattu G.S.[1,2,6*]

[1]Department of Urology, University of Michigan, Ann Arbor, MI.

[2]Rogel Cancer Center, University of Michigan, Ann Arbor, MI.

[3]Department of Internal Medicine, Division of Hematology/Oncology, University of Michigan, Ann Arbor, MI.

[4]Department of Surgery, University of Michigan, Ann Arbor, MI.

[5]Michigan Center for Translational Pathology, University of Michigan, Ann Arbor, MI.

[6]Department of Urology, Medical University of Vienna, Vienna, Austria.

[7]Department of Pathology, University of Michigan, Ann Arbor, MI

Running title: Platelet PD-L1 and cancer

Supplemental Figure 1

a

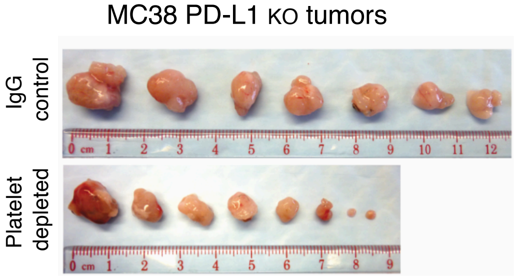

b

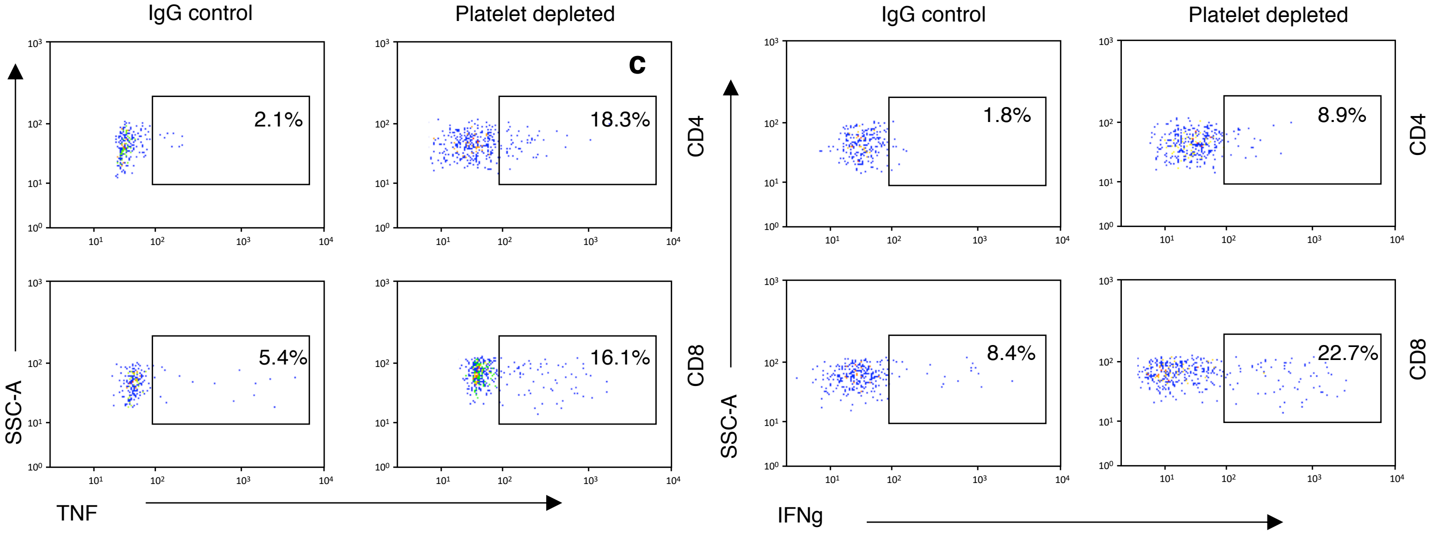

c

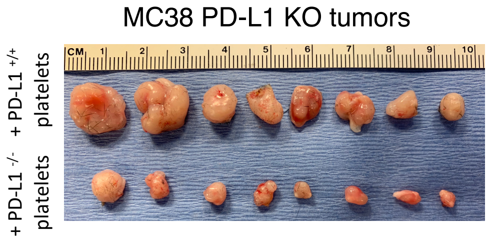

d

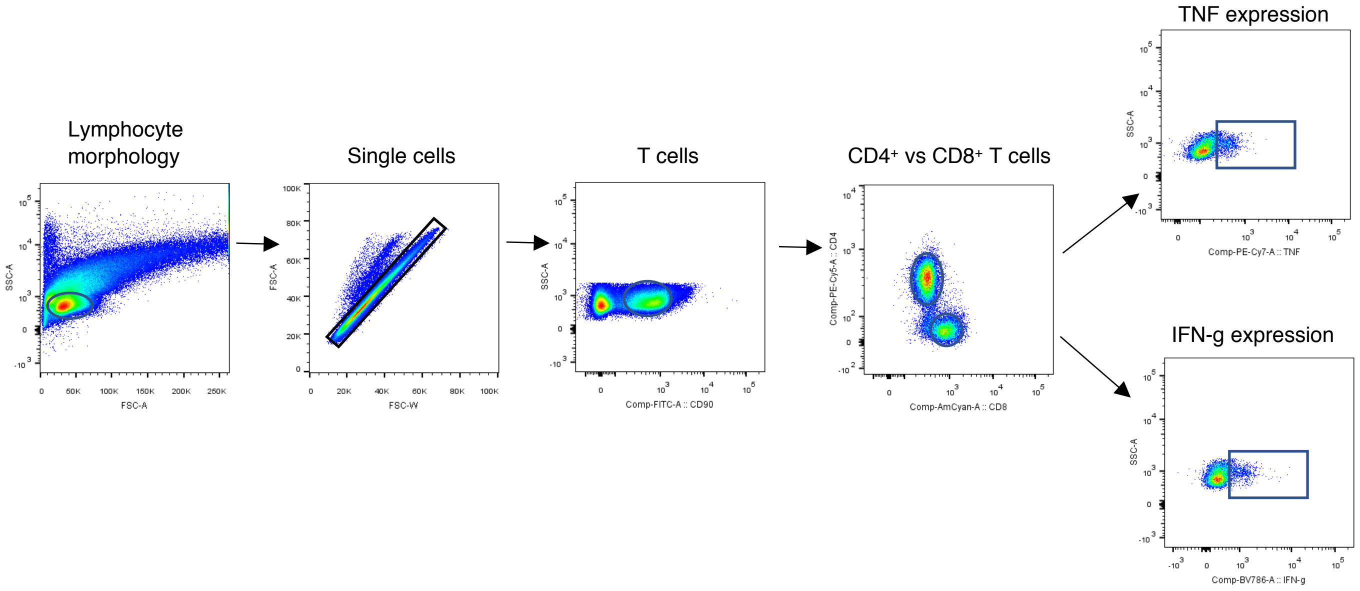

Supplemental Figure 2

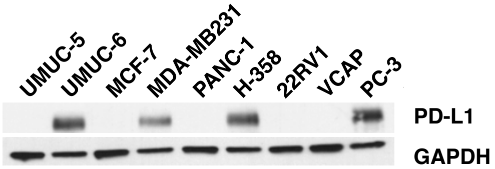

Supplemental Figure 3

a

cancer cells + platelets

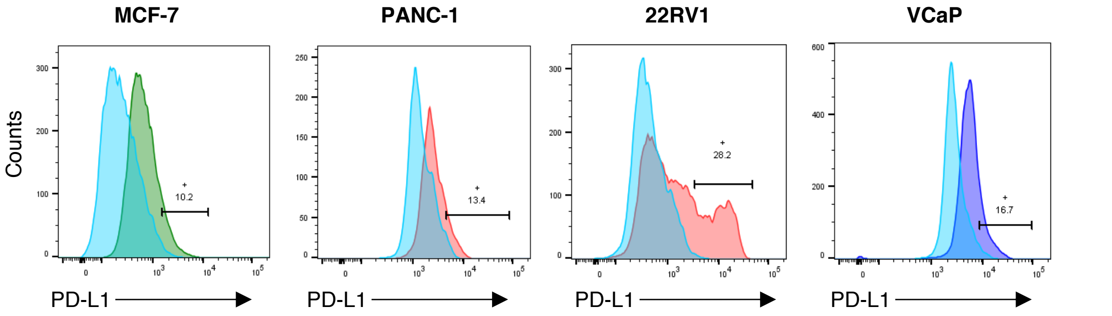

Blue = no platelets added

Green = after incubation w/ platelets

Blue = no platelets added

Red = after incubation w/ platelets

Blue = no platelets added

Red = after incubation w/ platelets

Blue = no platelets added

Purple = after incubation w/ platelets

b

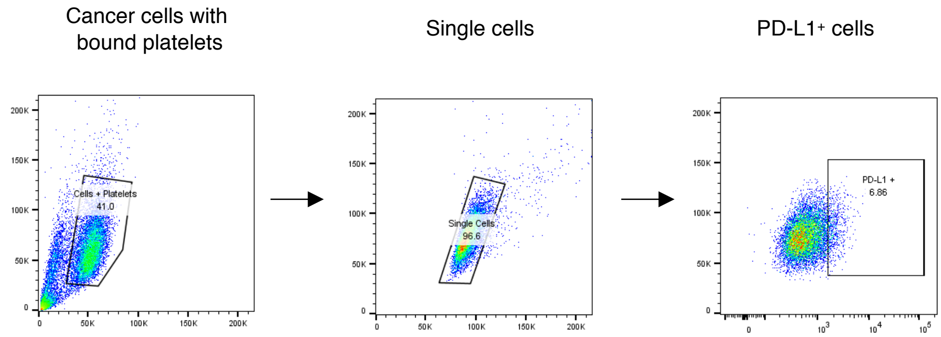

c

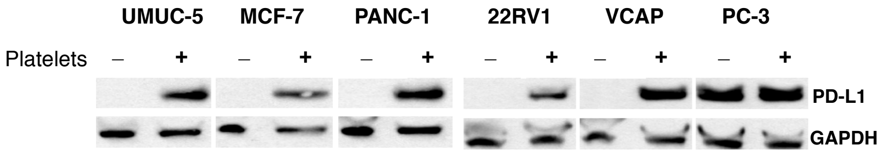

Supplemental Figure 4

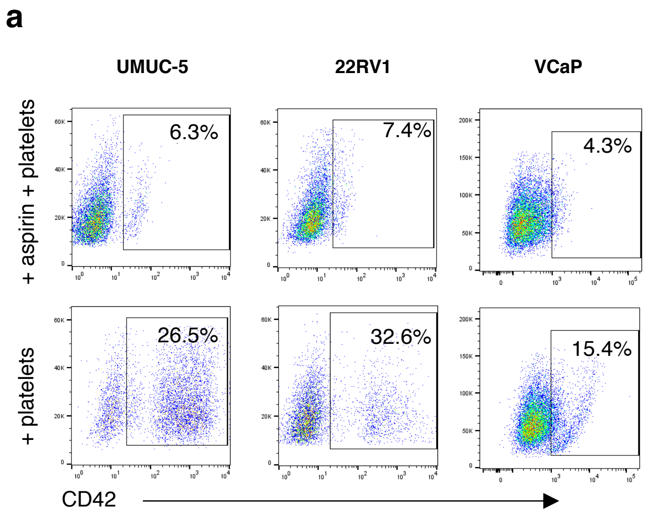

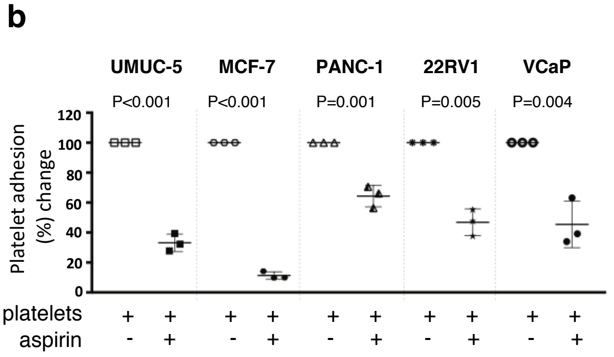

Supplemental Figure 5

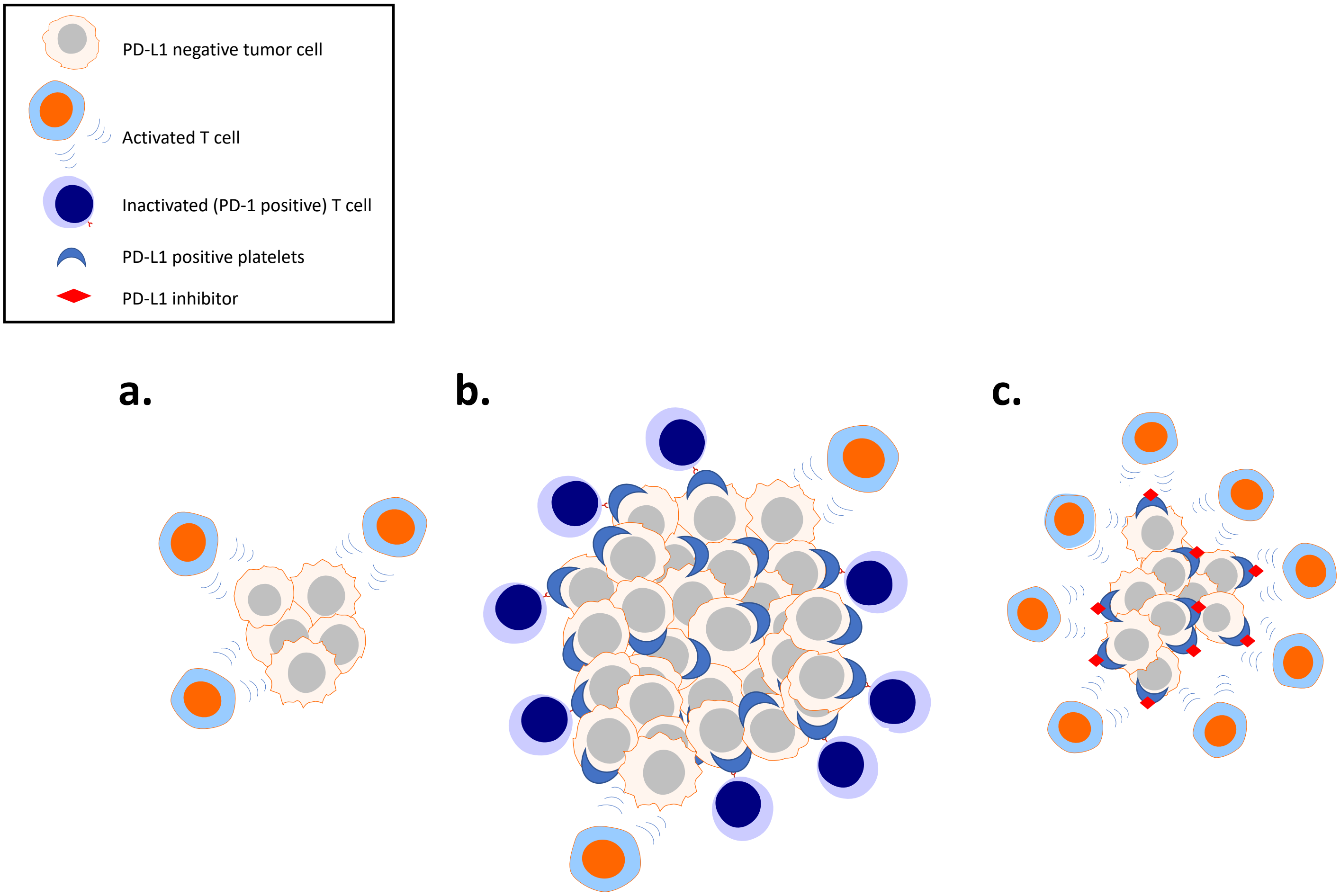

Figure 1 D

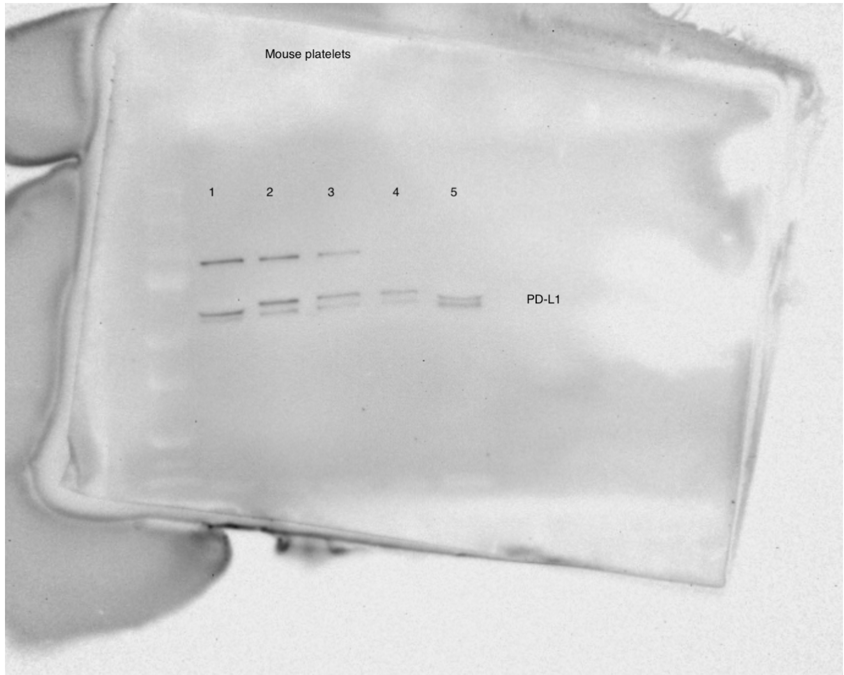

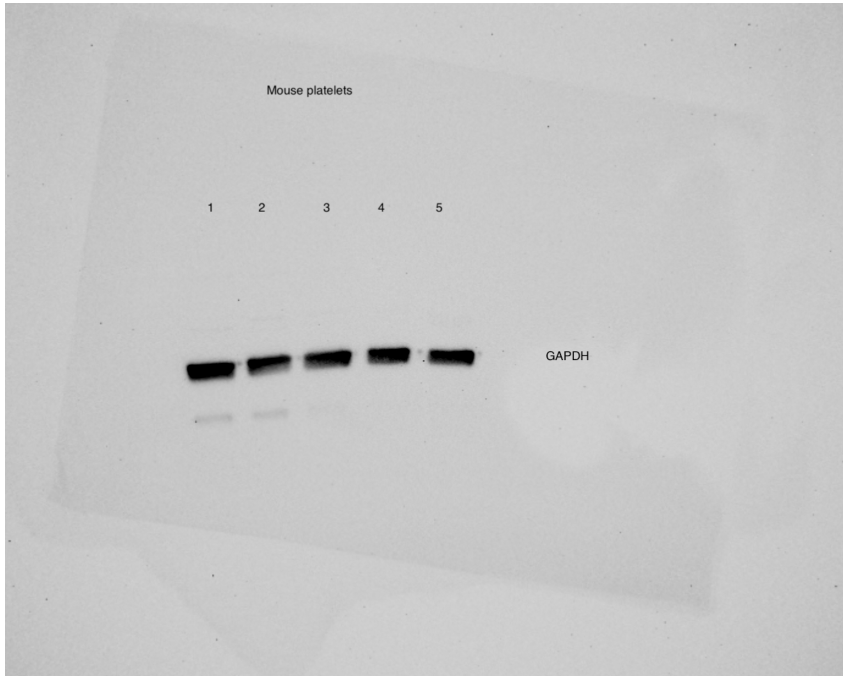

Figure 2 A

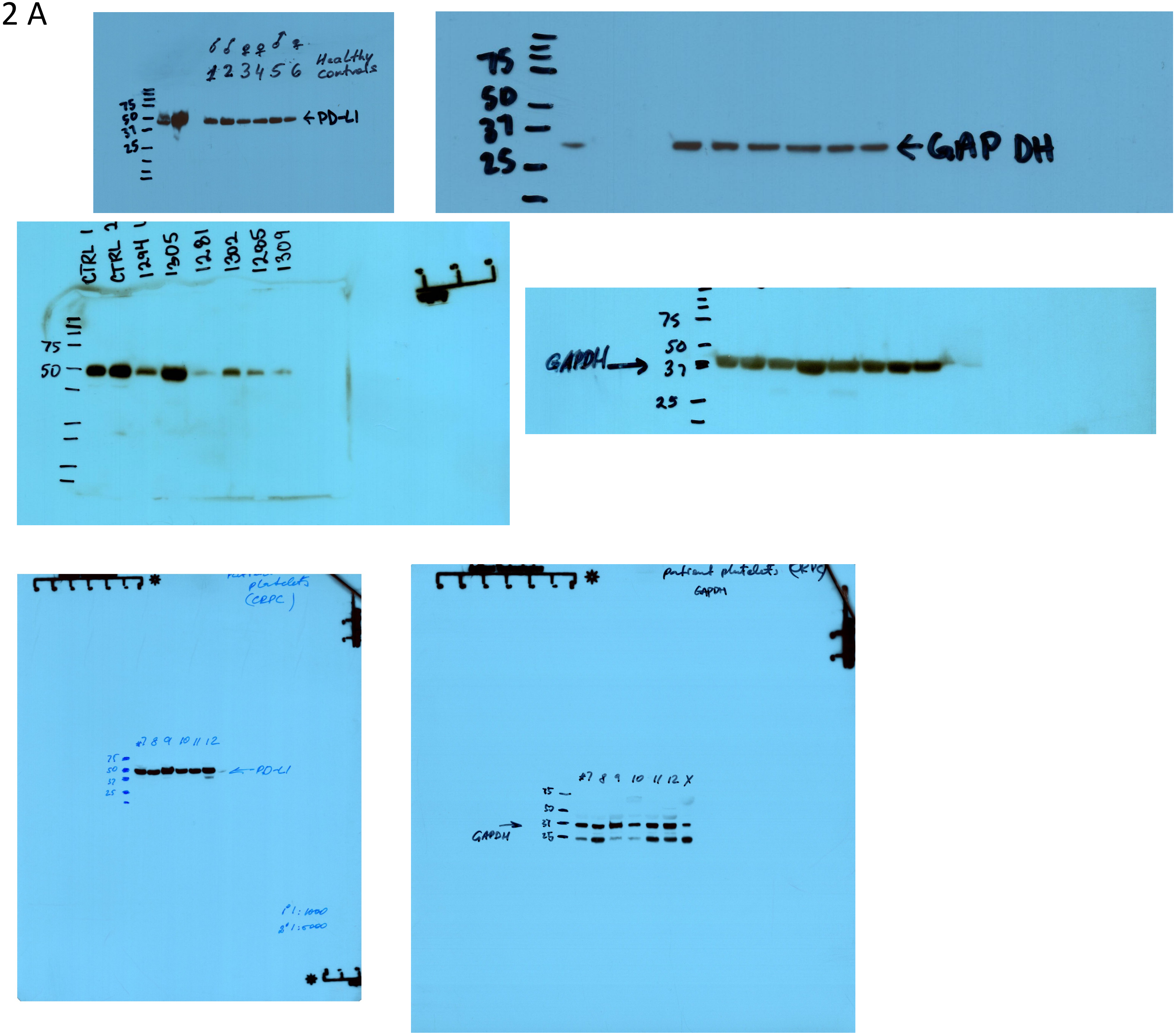

1 2 3 4 5 6
Healthy controls
75
50
37
25
PD-L1
GAPDH
CTRL 1
CTRL 2
1294
1305
1281
1302
1205
1309
patient platelets (CRPC)
#7 8 9 10 11 12
1°1:1000
2°1:5000
#7 8 9 10 11 12 X

Supplemental Figure 2

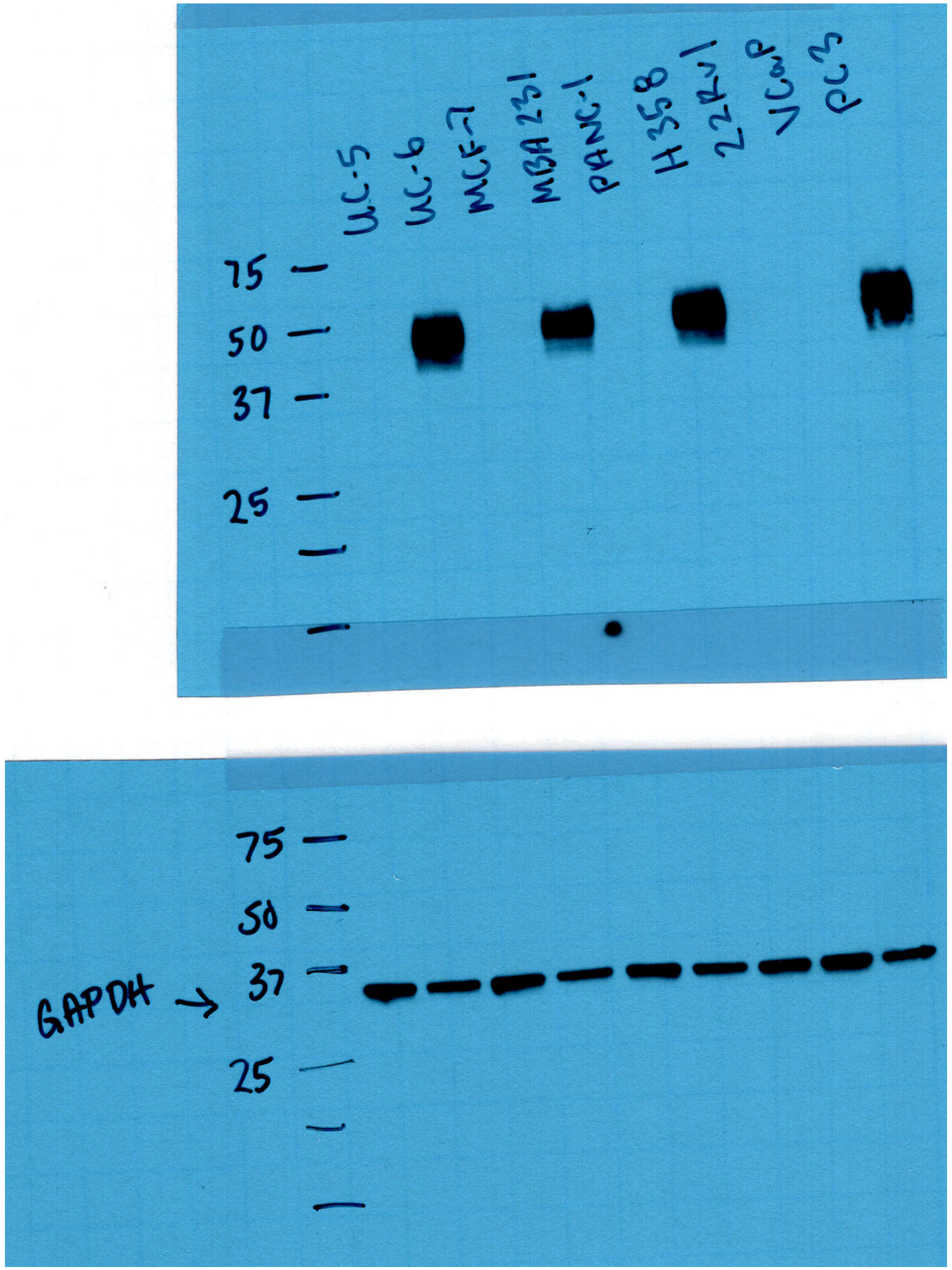

Supplemental Figure 3 C

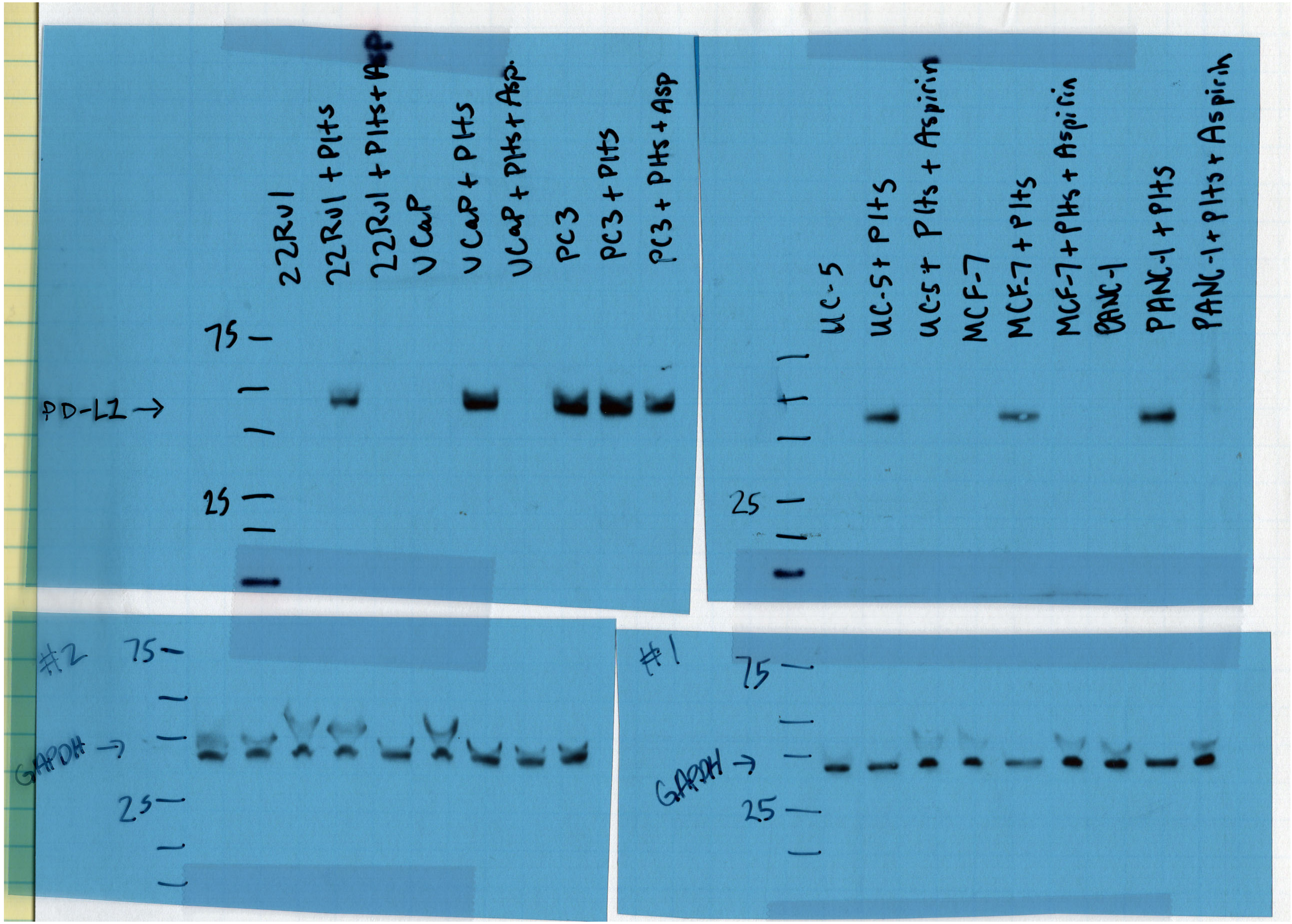

Supplement: Supplementary file 2 — Supplementary Figures. [file 41598_2020_76351_MOESM2_ESM.pdf]
